# Hybrid Data Fusion DBN for Intelligent Fault Diagnosis of Vehicle Reducers

**DOI:** 10.3390/s19112504

**Published:** 2019-05-31

**Authors:** Tianfan Zhang, Zhe Li, Zhenghong Deng, Bin Hu

**Affiliations:** 1 School of Automation, Northwestern Polytechnical University, Xi’an 710072, China; alitasoft@hotmail.com (T.Z.); dthree@nwpu.edu.cn (Z.D.); BinHu201205@hotmail.com (B.H.); 2 College of Economics and Management, Hubei Engineering University, Xiaogan 432100, China

**Keywords:** fault diagnosis, hybrid data fusion, mixed-precision training, hybrid deep belief nets, vehicle reducer

## Abstract

Given its importance, fault diagnosis has attracted considerable attention in the literature, and several machine learning methods have been proposed to discover the characteristics of different aspects in fault diagnosis. In this paper, we propose a Hybrid Deep Belief Network (HDBN) learning model that integrates data in different ways for intelligent fault diagnosis in motor drive systems, such as a vehicle drive system. In particular, we propose three data fusion methods: data union, data join, and data hybrid, based on detailed data fusion research. Additionally, the significance of the fusion is explained from the energy perspective of the signal. In particular, the appropriate fusion methods and data structures suitable for model training requirements can help improve the accuracy of fault diagnosis. Moreover, mixed-precision training is used as a special fusion method to further improve the performance of the model. Experiments with the datasets obtained from the simulation platform demonstrate the superiority of our proposed model over the state-of-the-art methods.

## 1. Introduction

**Figure 1:**
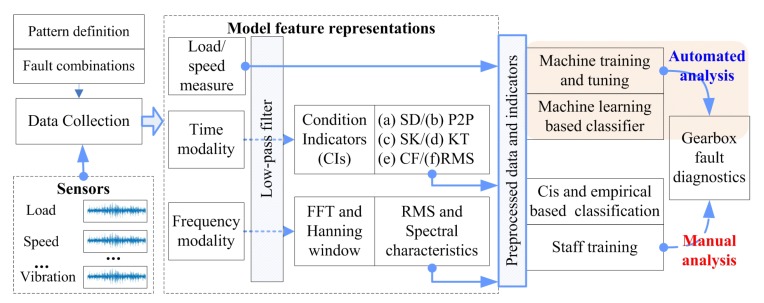
Comparison of the basic fault diagnostics system.

Rotary transmission equipment such as gearboxes are widely used in vehicles, automobiles, and other industrial equipment [1]. It is easy to cause machine strain and have serious consequences because of the accumulation of repetitive actions [2]. This “repetition” also provides an important reference for the fault diagnosis of such devices: one or more of the characteristics exhibited by the fault are enhanced by this “repetitive” process. There are two important issues that need to be addressed: first, how to determine the cycle of this “repetitive” process; second, how to determine the characteristics of the fault and establish a suitable evaluation method based on the feature. The Time Synchronous Averaged (TSA) [3] approach is one of the most widely utilized signal processing techniques to extract a periodic waveform from noisy signals of rotating machines and is an important method in time domain signal processing that can solve the first problem. The second problem is much more complicated, mainly because the fault may present different features under different viewing angles. According to different Condition Indicators (CIs), a series of methods is generated, for example the Standard Deviation (SD), Peak-to-Peak (P2P), Skewness (SK), Kurtosis (KT), Condition Factor (CF), and Root Mean Square (RMS) [4,5], as shown in Figure 1. Vibration signals are the focus of attention in this type of research [6,7] because the acquisition is easier and the characteristics are more obvious [5]. From an energy perspective, the fault signal is periodic (recurring), and the cumulative strength of the signal is continuously increased with the periodic operation of the gearbox. These considerations make the TSA-based CI analysis method an important diagnostic method [3].

The reliance on professional knowledge and expert experience and the immature fusion of multi-feature complex conditions limit the study of such empirical methods. Intelligent diagnostic methods have developed rapidly in recent years because of the ability to mine hidden fault features automatically from a variety of signals while relying less on expert experience [8]. A variety of methods include the Auto-Encoder (AE) [9], Restricted Boltzmann Machines (RBMs) and their variants, the Deep Belief Network (DBN) and Deep Boltzmann Machines (DBMs), as well as Convolutional Neural Networks (CNNs), Recurrent Neural Networks (RNNs) and Deep Learning (DL) [10]. In addition, this makes multisensor data fusion a key issue in intelligent diagnostic methods, since it provides an inexperienced “foolproof” signal fusion method and feature mining method [10,11,12]. In a recent study, the Denoising Auto-Encoder (DAE) [1,13] and Stacked Denoising Auto-Encoder (SDAE) [14,15,16] represented a type of development trend of DL-based machine health monitoring methods.

However, difficulties and challenges still exist [8,11]: First, existing research focuses on the impact of data fusion on the results, without explaining the meaning of data fusion based on the nature of the data itself (e.g., from the perspective of CIs) and how this fusion affects the learning model. We can call this method that does not consider the theory of fault diagnosis a “lazy model”. Second, such research tends to use more sensors to obtain more data to improve the accuracy of model diagnosis, while ignoring the conditional constraints in practical applications. More data means increased hardware requirements, power consumption, and computational latency.

In this paper, we propose an HDBN learning model that integrates data in different ways for intelligent fault diagnosis in motor drive systems, such as a vehicle drive system. In particular, we identify the physical meaning and specific methods of data fusion as two key parts: **(1) three basic data fusion methods; and (2) mixed-precision training.** In detail, data fusion does not mean simply “snaping” multiple and diverse data together, ignoring basic and physical meanings. In addition, it makes sense to reuse data from a variety of angles, for example mixed-precision training that can enhance the model accuracy without having to incur more cost to obtain more data. Our work makes the following contributions.

- We have established an efficient fault diagnosis model, namely, HDBN, which is based on an energy perspective that focuses on data fusion for signal analysis and fault diagnosis of rotating devices.
- We explain the significance and role of data (signal) fusion from a physical perspective, and three basic fusion methods are proposed: data union, data join, and data hybrid.
- We present a hybrid precision training algorithm to improve the overall performance of our proposed model without collecting more data.

The remainder of this paper is organized as follows. Section 2 briefly reviews the literature. Section 3 describes the intelligent HDBN fault diagnosis model and explains the mechanism and role of the energy perspective in fault diagnosis based on the TSA and CI methods.

Then, the HDBN fault diagnosis model is established based on the energy function. The major contributions are introduced in Section 4, emphasizing the fusion process and the physical meaning of its signals that have been carefully studied in data fusion as a key issue in HDBN. Furthermore, mixed-precision training is used to further enhance the performance of the model. In Section 5, we present a complete experimental platform and unmanned vehicle application case, and we compare our proposed method with existing intelligent diagnostic approaches. Section 6 concludes this paper.

## 2. Related Works

Machine Health Monitoring Systems (MHMS) form an integral part of the new industrial era. The Industrial Internet of Things (IoT) and data-driven techniques have been revolutionizing manufacturing by enabling computer networks to gather the huge amount of data from connected machines and turn the big machinery data into actionable information [17,18,19]. Data-driven MHMS is a key component of modern manufacturing systems Two major research topics are related to this work, including multi-condition fault diagnosis methods and intelligent diagnostic methods and data fusion research for machine learning methods. Whether it is a classic or a machine learning method, these approaches have similarities in the data acquisition and preprocessing stages, which are still in the time domain or frequency domain [5]. The comparison of a basic fault diagnostics system is shown in Figure 1.

Traditional data-driven MHMS relies on the experience of experts. That is, it is necessary for a professional to select appropriate features manually according to specific problems and perform feature extraction and training at appropriate angles such as the frequency domain and time domain. IMF [20] tools based on Fast Fourier Transform (FFT) and Hilbert–Huang Transform (HHT) [21], DB-KIT [22,23,24] are typical application tools for this type of research. These tools help us identify and capture characterization data and then use some statistical methods such as [6] to provide a review of the earlier literature on condition monitoring of a gearbox based on vibration signals, including the SD, P2P, and SK methods. The work in [2,8] updated this type of study and included SD, P2P, SK, and other methods. Shallow learning methods such as Support Vector Machines (SVM) [25], Naive Bayes (NB) [26], and logistic regression [27] are also common methods. The time domain method has made some progress in early research as a simple basic method [28]. Converting a time domain signal into the frequency or another domain suppresses noise while highlighting features, because background noise is also included in the time domain signal. The work in [7] expressed the resonance signal caused by the fault through Amplitude Modulation and Frequency Modulation (AM-FM) processes and derived the explicit equation of the Fourier spectrum. The work in [5] proposed a time-frequency analysis method based on an Ensemble Local Mean Decomposition (ELMD) and FKthat effectively separates fault features from non-Gaussian noise and ambient noise. The work in [29] proposed a mixed H_ / $H_{\infty }$ fault detector design method based on the Linear-Parameter-varying (LPV) model for fault detection of a steering actuator in an Electric Ground Vehicle (EGV). Moreover, the work in [3] presented a new TSA-based method using a single piezoelectric strain sensor for Planetary Gearbox (PGB) fault diagnosis.

However, it is difficult to design appropriate features and perform feature selection. Manually designing features for a complex domain requires a great deal of human labor and cannot be updated on-line. These methods attempt to define fault features for one or a specific type of condition from different perspectives (especially the vibration signal as an energy view) based on experience, as we have introduced in the Introduction. As we all know, the complex and noisy working condition hinders the construction of physical models, which make the modeling of complex dynamic systems very difficult [29]. There is a deepened reliance on expert knowledge and experience in order to obtain strong fault characteristics and to achieve more adaptability, which is not always available.

Deep learning-based MHMS provides a bottom-up solution. A deep neural network with multiple layers of nonlinear transformation is constructed to extract hierarchical representations from input data. The conversion of the input value to the output value takes place in each layer. From a data-driven perspective, the “weak” feature that contains the original “non-dominant” will gradually become “strong” after multiple layers of training until it is sufficient to represent the identity of the object clearly such as fault classification. Compared to conventional data-driven MHMS, DL-based MHMS does not require extensive human labor and knowledge for hand-crafted feature design. Therefore, DL-based models can be applied to address machine health monitoring in a very general manner. The data and feature fusion methods provided by machine learning greatly reduce the dependence on experience and have enabled more researchers to enter the field of drive fault diagnosis [30]. Deep learning is a novel machine learning method based on multiple nonlinear transformations that can be used to extract deep features from raw data automatically.

In general, the deep learning model is mainly divided into auto-encoders [31,32], deep belief networks [33,34], deep Boltzmann machines [28], convolutional neural networks [29,35], Deep Neural Networks (DNN) [10], recurrent neural networks [30], and various variants and optimized versions derived therefrom. Deep learning generally refers to a network with a multi-layer structure, more like an artificial neural network. We try to learn the hierarchical representation of data through multiple nonlinear processing layers [36]. In the deep learning that originated from the “perceptron”, the most basic structure is to combine multiple perceptrons to form a multi-layer perceptron [37]. A convolutional neural network is obtained by adding a cortical structure similar to human vision. The other category originated from the “Peltzman machine based on graph model” [38]. The former belongs to supervised learning and outputs training networks according to expectations; the latter is an unsupervised learning that trains networks only based on specific training data.

The automatic encoder consists of two parts, an encoder and a decoder, as a feedforward neural network. It is designed to learn a new representation of the data by attempting to reconstruct the input data [32]. The encoder accepts input *x* and converts it to a hidden representation *h* by nonlinear mapping. To prevent the learned transformation from being the identity one and to regularize auto-encoders, the sparsity constraint is imposed on the hidden units [9,39]. In order to overcome the effects of noise interference in the operating environment and data loss caused by random network time delay [40], the addition of denoising AE takes a corrupted version of data as input and is trained to reconstruct/denoise the clean input *x* from its corrupted sample $\tilde{x}$, achieving better adaptability. Since the automatic encoder can be trained in an unsupervised manner, automatic encoders, especially Stacked Denoising Automatic encoders (SDAs), can train the model by initializing the weight of the Deep Neural Network (DNN) to provide effective pre-training [41]. The work in [42] proposed a novel energy-fluctuated multiscale feature mining approach based on a Wavelet Packet Energy (WPE) image and DCN for spindle bearing fault diagnosis.

RBM is a two-layer neural network as a Markov random field. A bipartite graph that consists of two groups of units including visible units *v* and hidden units *h* under the constraint. DBN and Deep Boltzmann (DBM) systems are derived from RBM. The DBN can be established by stacking multiple RMBs. Similar to SDA, DBNs can be trained in a greedy, layered, unsupervised manner. DBM can be seen as a deep-structured RBM. The main difference between DBN and DBM is that DBM is a completely undirected graphical model, while DBN is a mixed directed/undirected graphical model. Unlike DBNs that can be trained layer by layer, DBM is trained as a joint model. Therefore, DBM training is computationally more expensive than DBN.

More data from multiple sensors means higher accuracy and adaptability due to the nature of deep learning methods. The work in [43] proposed a comprehensive review of the data fusion state-of-the-art approach, exploring its conceptualizations, benefits, and challenging aspects, as well as existing methodologies. The work in [34] proposed a two-layer Sparse Auto-Encoder-Deep Belief Network (SAE-DBN), and the work in [44] proposed an Adaptive Neuro-Fuzzy Inference System (ANFIS) in which multiple accelerometers were used. The work in [45] combined a multivariate orthogonal space transformation and vectorized time-series models into a system equipped with multisensor networks to implement a residual-based fault monitoring system. The work in [46] proposed an unsupervised feature extraction method based on Greedy Kernel Principal Component Analysis (GKPCA) under multidimensional unlabeled signal conditions, which has improved monotonicity, robustness, and computational speed performance. The study by [47] presented a new probabilistic nonlinear feature selection and fusion method, named Probabilistic Kernel Factor Analysis (PKFA), in order to solve the feature selection and fusion problem in machinery condition monitoring. DBN is a good fault feature mining model in which [48] combined spectral data acquired from three identical acceleration sensors based on DBN and established the ball screw degradation recognition method. The work in [49] achieved rolling bearing fault diagnosis based on DBN through multiple values obtained by multiple different sensors. The RNN can generate and address the memory of an input mode sequence of any length. It can be built in a directional loop different from the basic neural network: the multi-layer perceptron can only map from the input data to the target vector; the RNN can in principle map the entire history of the previous input to the target vector and allow the previously entered memory to be stored in the internal state of the network, thus combining with LSTM and applying MHMS [50].

Multisensor data fusion remains a challenging issue [51], although some good research progress has been made. Current research is more focused on how to perform efficient feature extraction, such as the above studies, while ignoring the methods, roles, and mechanisms of data fusion. Another challenge is the selection of different fusion levels. Similarly, different fusion levels have their own advantages and disadvantages, and the suitable ones for different fault diagnosis tasks are usually different [52]. Selecting an optimal fusion level for a specific fault diagnosis task always requires domain expertise, prior knowledge, and human labor [43]. Therefore, we have proposed the HDBN method in order to solve these problems.

## 3. Methodology

**Figure 2:**
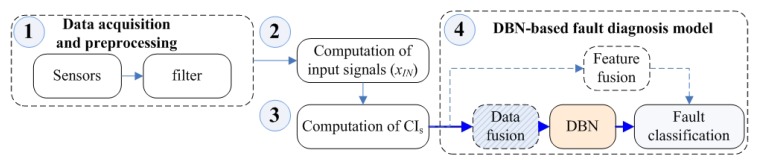
The basic model of TSA.

The HDBN establishment process is divided into two parts: the basic fault diagnostics system and the hybrid data fusion process. Among these parts, the establishment process of the basic model can be divided into three steps, as shown in Figure 2.

First, it was necessary to preprocess the acquired raw data, such as vibration signals. In addition, the data were filtered by a suitable low-pass filter [3]. Second, the TSA signal, residual signals, and other input signals were computed based on tachometer signals. Finally, the condition indicator was calculated. Then, the DBN method was used instead of the experience-dependent method, and we could define the basic diagnostic model.

### 3.1. TSA-Based CI Diagnostic Method from an Energy Perspective

The basic idea is that when the motor is running at a constant speed, its periodic signals (such as fault signals) are boosted to a higher level and are clearly distinguished from the noise signal. Assuming the total number of *N* observed periods, the TSA of x(t) can be expressed as [53]:

(1) $$x_{TSA}=\frac{1}{N}\sum_{r=0}^{N-1}x(t-rT_{R})$$

where *r* is the index of the periodic signal, $T_{R}$ is the time of a cycle, and $\Delta t=t-rT_{R}$. Basically, TSA chops up the raw sensor signal into multiple single-revolution signals. Then, each of the revolution signals is resampled to have the same number of sample points in one revolution. Next, the final periodic signal is obtained by averaging the resampled signals. After TSA is computed, any kind of fault diagnostic CI can be evaluated [54,55].

There are a variety of definitions of CIs, as shown in Figure 1. Each type of CI can be computed using different input signals. The energy operator (EO) [4] is defined as the residual of the autocorrelation function as follows:

(2) $$\mathrm{for}\;i=2,3,\cdots ,N-1\left\{x_{EO,i-1}=x_{TSA,i}^{2}-\left(x_{TSA,i-1}\;\cdot \;x_{TSA,i+1}\right)\right\}$$

where $x_{EO,i}$ is the *i*th element of EO data. In this way, the type of fault can be determined by analyzing the residuals of different features.

As another point of view, the Fourier Transform (FT) is a classical vibration fault analysis method. By changing the vibration signal from the time threshold to the frequency domain, this energy view can be expressed more intuitively. The result converted by fast Fourier Transform (FFT) equation $X\left(f\right)=\int_{-\infty }^{\infty }x\left(t\right)e^{-i2\pi ft}dt$ can be found in Section 5.2. The fault’s main feature component is highlighted by the FFT to convert the signal that is decomposed into multiple segments into a spectrum [56]. Then, a comparison is performed using a waterfall graph. This feature component is further enhanced if iterated using the TSA method described above. However, there is still a large number of lower amplitude components in the figure, which will also affect the actual effect of TSA.

### 3.2. Basic DBN Model

The TSA-based multiple CI analysis method provides us with a feasible diagnostic method. However, there are two problems: The data (signal) segmentation has certain difficulties if the period of the signal and its starting point are not clear and there is lack of expert experience. Another issue is how to fuse multiple data or conditions to enhance fault characteristics. DBN does not pay attention to the segmentation process of the precise period in TSA, as long as the dimension after data segmentation is appropriate (easy to store and train). Additionally, this will be discussed in Section 5. For the fusion problem of multiple CIs, from different perspectives, it can be a data fusion problem (using multiple CIs as training conditions to input to the DBN) or a feature fusion problem (i.e., CIs as a result of feature mining).

In this article, we will focus on the data fusion problem of CIs.

Another reason for choosing DBN as the base model is that it exhibits better performance under low speed regulation conditions in pre-training.

*(1) RBMs:* DBN is a multilayer neural network consisting of a series of RBMs that are stacked [57].

**Figure 3:**
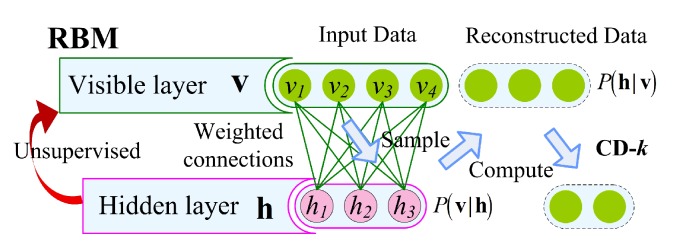
The basic model of RBMs.

The structure of RMB is a bipartite graph, that is the nodes in the layer are not connected; as shown in Figure 3, where the first layer is the input node $V_{v}$, and its state space is $\left\{0,1\right\}$ or a real number R. The second layer is the hidden node $V_{h}$, and the state space is $\left\{0,1\right\}$. W is the connection weight coefficient matrix of $V_{v}$ and $V_{h}$.

Each of these nodes can take different states. The state of this model is also determined when the state of each node is determined and refers to the degree to which the model takes the state; moreover, the model is evaluated by the energy function.

When the state space of $V_{v}$ is $\left\{0,1\right\}$, the energy function is defined as:

(3) $$E\left(v,h\right)=-\sum_{i,j}v_{i}W_{ij}h_{j}-\sum_{i\in visible}a_{j}v_{j}-\sum_{j\in hidden}b_{j}h_{j}$$

*(2) k-step Contrastive Divergence Method (k-CDM):* We used the k-CDM training method [48] because the default Gibbs sampling RBM training method is less efficient [49]. There are two one-step methods “binary to binary” and “Gaussian to Gaussian”. The model of CD-1 (binary to binary) is as follows:

Above all, there is the input node $V_{v}\in \left[0,1\right]$, and the loss function is defined as:

(4) $$Loss=\sum {\left(v-p_{v^{\prime }}\right)}^{2}$$

In the second layer, regarding p(h|v), if $p_{h}>\mathrm{rand}\left(0,1\right)$, then h=1; otherwise, h=0. The reconstructed data are returned when the calculation is complete, and p(v|h) can be determined by calculation. When the data are passed back to the hidden layer, the output is:

(5) $$\left\{\begin{array}{c} p_{h}=\mathrm{sigmoid}\left(v\;\cdot \;W+b\right)\\ p_{v^{\prime }}=\mathrm{sigmoid}\left(h\;\cdot \;W^{T}+a\right)\\ p_{h^{\prime }}=\mathrm{sigmoid}\left(p_{v^{\prime }}\;\cdot \;W+b\right) \end{array}\right.$$

The calculations of $\Delta W^{e}$, $\Delta a^{e}$, and $\Delta b^{e}$ are:

(6) $$\left\{\begin{array}{c} \Delta W^{e}=\left(v^{T}\;\cdot \;p_{h}-p_{v^{\prime }}^{T}\;\cdot \;p_{h^{\prime }}\right)/n\\ \Delta a^{e}=\sum \left(v-p_{v^{\prime }}\right)/n\\ \Delta b^{e}=\sum \left(p_{h}-p_{h^{\prime }}\right)/n \end{array}\right.$$

Then, $W^{e+1}$, $a^{e+1}$, and $b^{e+1}$ can be calculated:

(7) $$\left\{\begin{array}{c} W^{e+1}=W^{e}+m\Delta W^{e-1}+r\Delta W^{e}-dW^{e}\\ a^{e=1}=a^{e}+m\;\cdot \;\Delta a^{e-1}+r\;\cdot \;\Delta a^{e}\\ b^{e=1}=b^{e}+m\;\cdot \;\Delta b^{e-1}+r\;\cdot \;\Delta b^{e} \end{array}\right.$$

In the model of CD-1 (Gaussian to Gaussian), the energy function is redefined as:

(8) $$E\left(v,h\right)=-\sum_{i,j}\frac{v_{i}}{\sigma_{i}}\frac{h_{j}}{\sigma_{j}}W_{ij}+\sum_{i\in visible}\frac{{\left(a_{i}-v_{i}\right)}^{2}}{2\sigma_{i}^{2}}+\sum_{j\in hidden}\frac{{\left(b_{j}-h_{j}\right)}^{2}}{2\sigma_{j}^{2}}$$

If the *K*-step is set to one-step, then $\sigma_{i}=1$, $\sigma_{j}=1$ and the Formula (5) in the process is changed to:

(9) $$\left\{\begin{array}{c} p_{h}∼N\left(v\;\cdot \;W+a,\sigma \right),h=v\;\cdot \;W+a\\ p_{v^{\prime }}∼N\left(h\;\cdot \;W^{T}+b,\sigma \right),v^{\prime }=h\;\cdot \;W^{T}+b\\ p_{h^{\prime }}∼N\left(v^{\prime }\;\cdot \;W+a,\sigma \right),h^{\prime }=v^{\prime }\;\cdot \;W+a \end{array}\right.$$

while the Formula (6) changes to:

(10) $$\left\{\begin{array}{c} \Delta W^{e}=\left(v^{T}\;\cdot \;h-v{{}^{\prime }}^{T}\;\cdot \;p_{h^{\prime }}\right)/n\\ \Delta a^{e}=\sum \left(v-v^{\prime }\right)/n\\ \Delta b^{e}=\sum \left(h-h^{\prime }\right)/n \end{array}\right.$$

**Figure 4:**
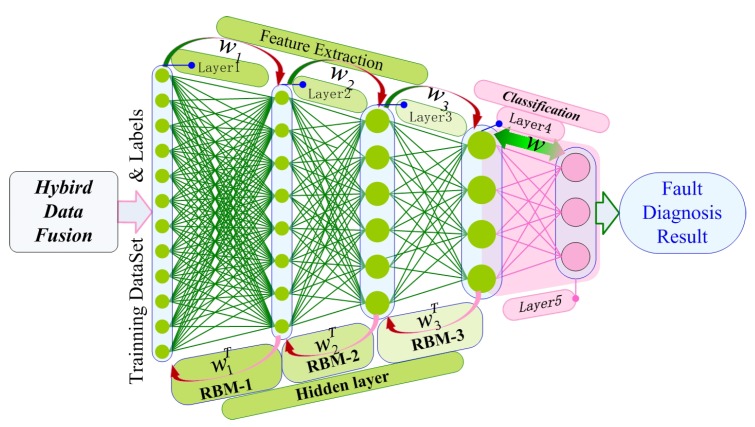
HDBN architecture.

*(3) Building DBN:* In this way, the DBN model is built by linking the RBM layers, as shown in Figure 4. Additionally, the HDBN model will be built after solving the hybrid data fusion problem.

## 4. Hybrid Data Fusion and Model Improvement

### 4.1. Pretreatment

**Figure 5:**
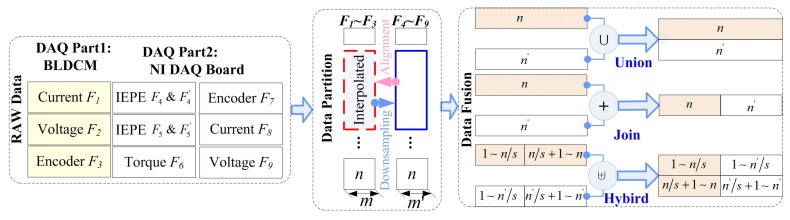
Hybrid data fusion structure.

Data fusion has an important impact on training models and fault classification. The specific process, methods, and impacts of data fusion have not been carefully studied, although many studies in the literature have proposed analytical models based on data fusion. Therefore, this paper carefully studies and elaborates three types of data fusion processes and their different effects, as shown in Figure 5. The approach is mainly divided into three parts: data preprocessing, data segmentation, and data fusion.

Different data sources can have a significant impact on data fusion and its outcome. Taking the experimental platform shown in Section 5 of this paper as an example, the data of eight sensors $F_{1}$∼$F_{8}$ wre obtained from two parts: the BLDCM and NI DAQ board. The sampling frequency and accuracy of the BLDCM are lower than those of the NI DAQ board. In addition, we have to preprocess different data separately because there are five different kinds of sensors.

The preprocessed data need to be processed by segmentation, labeling, etc., before being sent to the model to form a dataset. Obviously, the length of the data is different due to the difference in sampling frequency. For example, both $F_{1}$ and $F_{4}$ are current data, but they have different lengths and need to be “aligned” before the merger. Using interpolation is appropriate, although downsampling also enables data alignment. However, sampling will greatly reduce the total number of samples, which directly affects the training accuracy. Moreover, they will be split into two types of datasets $DS_{1}\left(m\times n\right)$ and $DS_{2}\left(m^{\prime }\times n^{\prime }\right)$, where $m^{\prime }\geq m$ and $n=n^{\prime }$ (interpolation or downsampling was used to facilitate calculation) for the appropriate data. Here, *m* and $m^{\prime }$ represent the number of rows of the set, and *n* identifies the column as the feature of the signal.

### 4.2. Hybrid Data Fusion Method

The three fusion methods **union**, **join**, and **hybrid** are proposed according to the source of the data.

*(1)* “*Union* (∪)” is a basic data fusion method, and even data of different kinds, such as frequency and current, can be combined to form a training set. For the two datasets with the same number of features, the (∪) can be defined when $n=n^{\prime }$ as:

(11) $$DS_{1}\left(m\times n\right)\cup DS_{2}\left(m^{\prime }\times n^{\prime }\right)=DS^{\prime }\left(\left[m+m^{\prime }\right]\times n\right)$$

In this way, if more samples (rows) are merged, it is beneficial to improve the accuracy of machine learning; thus, it is the most common data fusion method in the existing research.

*(2)* “*Join*” (⊕) operations are suitable for the same type of data, such as vibration signals from different installation locations. The merging operation is suitable for the same type of data, such as vibration signals from different mounting positions. From a physical point of view, this is equivalent to merging the same but different phase signals in a single sample. For example, in this paper, the installation angles of the two vibration sensors are $90^{\circ }$ with each other. The **⊕** operations can be defined when $m=m^{\prime }$ as:

(12) $$DS_{1}\left(m\times n\right)⊕DS_{2}\left(m^{\prime }\times n\right)=DS^{\prime }\left(m\times \left[n+n^{\prime }\right]\right)$$

Thus, the number of features (cols) of a single sample is increased. The total number of samples (rows) has decreased. This fusion approach may reduce recognition accuracy based on machine learning experience.

*(3)* “*Hybrid*” (⊎) operations can be defined by combining the two fusion methods ∪ and **⊕**. This is similar to the approach that we used in data segmentation. The main difference is that the segmentation granularity is reduced to n/s, and *s* is the partition coefficient with s∈[1,n]. Then, ⊕ fusion is performed on the new sample. Finally, more samples (especially a dissimilar sample) are then fused by the ∪ fusion method. Thus, ⊎=∪ when s=1 and ⊎=⊕ when s=n. It is possible to flexibly adjust the specific fusion mode and degree through the adjustment of *s*. Additionally, s=2 is the default setting. The advantage of this approach is that we can both increase the sample size and optimize and further enhance the sample in the physical sense. The ⊎ operations can be defined as:

(13) $$DS_{1}\left(m\times n\right)⊎DS_{2}\left(m^{\prime }\times n^{\prime }\right)=DS^{\prime }\left[\begin{array}{cc} 1∼\frac{n}{s} & 1∼\frac{n^{\prime }}{s}\\ \frac{n}{s}+1∼n & \frac{n^{\prime }}{s}+1∼n^{\prime } \end{array}\right]$$

The segmentation and fusion of such datasets is only in the physical sense. In addition, this process does not change the essential characteristics of the data. It is equivalent to observing the target (dataset) from different perspectives in order to better discover its characteristics. This approach leads to two basic results: the deep learning approach helps to mine deep, implicit features from the data without having to rely too much on expert experience because experts understand which perspectives are easier to observe. More data means that more distinctive features can be observed and learned to improve the accuracy of the model; even if the same sensor acquires the same signal at different locations, it will help the model improve. This is also the root cause of most intelligent diagnostic methods that choose deep learning models or using multisensor fusion.

In particular, the FFT method converts the signal from the time domain to the frequency domain, at which point its essential characteristics have changed and $F_{7}^{\prime }=\mathrm{FFT}\left(F_{7}\right)$, $F_{8}^{\prime }=\mathrm{FFT}\left(F_{8}\right)$. Moreover, new features may be introduced to the model without adding new samples. This approach is analogous to two simple forms of carbon—graphite and diamond—although they are homologous, but different in traits.

We can make the following assumptions based on the above analysis:

**Assumption** **1.** *It is possible to improve the accuracy of the model within a limited range by using the ∪, ⊕ or ⊎ methods alone.*

**Assumption** **2.** *Limited enhancements or even side effects occur if the ⊕ method is used. This is because the dimension of the data is increased, but the total number of samples is reduced. In addition, the cost of model learning has been improved.*

**Assumption** **3.** *Larger enhancements can be expected by fusing a variety of different features, such as $\left(F_{7}\cup F_{8}\right)⊎\left(F_{7}⊎F_{8}\right)$, without reducing the number of samples.*

**Assumption** **4.** *Larger boosts can be expected by introducing data of a different nature, including multisource sensor data or changed data such as $F_{7}^{\prime }$ and $F_{8}^{\prime }$ or $F_{1}$∼$F_{6}$.*

### 4.3. Mixed-Precision Training

**Figure 6:**
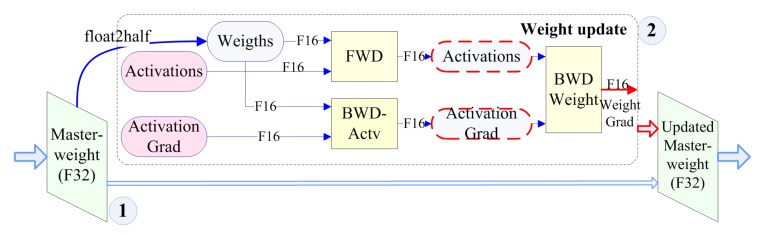
Mixed-precision training model.

The mixed-precision training model is constructed to further explore the potential of existing data and enhance the above model [58] as shown in Figure 6. First, the original samples can be classified into Float 16 (F16) and Float 32 (F32) according to the storage format and precision. The F32 with higher precision is called the master-weight. F16 data are converted by F32 through the float2half function. Then, in the second part, we activate the function calculator to calculate its weight level and fuse it with the master-weight to obtain the updated weight. This mixed-precision training has two functions: F16 low-precision data help speed up the pre-training process and provide directional guidance for feature mining; and the training (weight) data used to update the master-weight help improve accuracy.

In addition, unlike this pure software approach, there are two sets of data from the higher precision NI DAQ system and the lower precision data from BLDC acquisition in our experimental system. This situation allows us to compare both software and physical (signal source) angles simultaneously.

**Assumption** **5.** *Although the effect may be limited, mixed-precision training is beneficial for improving the accuracy of the model.*

## 5. Experimental Setup

### 5.1. Experimental Platform and Fault Seeds

*(1) Experimental platform:*

**Figure 7:**
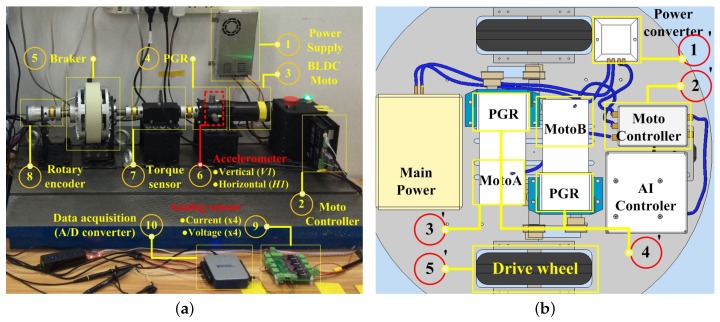
Experimental device architecture of the system. (**a**) Experimental architecture. (**b**) A vehicle instance designed by the author.

The experimental system is composed of ten parts. Figure 7a displays the PGB test rig used to collect the data under different gear health and operating conditions.

① Power supply: Converts 220 V AC power to 24 V DC with constant voltage mode. ② BLDC Motor controller. It has the ability to measure system current and voltage through an integrated 8-bit sensor. In addition, based on the CAN bus, the command and monitoring data are exchanged through the “COM-CAN” converter at a sampling rate of 50 Hz. ③ Brushless direct current motor (BLDCM), rated power 24 V 5.6 A, 3000 RPM; output torque 0.42 n/m. In addition, a 1000-pulse/s encoder was integrated for measuring motor speed. ④ A commercially-available single-stage PGB with a 10:1 speed.

Additionally, a price below $25 makes it less likely to achieve good enough performance. ⑤ A magnetic powder brake for simulating loads. ⑥ Integrated Electronics Piezoelectric (IEPE)-type of accelerometer. Two were glued on the housing of the ring gear. One was in the vertical position (V1), and the other was in the horizontal position (H1) in order to accurately capture their vibration signals. ⑦ Dynamic torque sensor. ⑧ Rotary encoder for obtaining output speed. ⑨ Four-way voltage and four-way current analog sensor. ⑩ Data acquisition card: NI UB-6002, 16 bit. Performs A/D conversion and transfer data to PC.

It is noteworthy that the above components may be adjusted. The actual vehicle system was limited by factors such as size, cost, and environment of use. For example, torsion sensors are difficult to deploy based on compact design considerations. Figure 7b displays a set of unmanned vehicle systems designed by the author team for use in agriculture. There is not enough space here to deploy additional large sensors such as torsion sensors. The system is composed of five parts from ${①}^{\prime }$–${⑤}^{\prime }$ in the experimental environment [40]. In addition, there are some differences here. In Section (1), the AC power source needs to be replaced with a power battery. For ⑧, it is necessary if the motor of ${⑤}^{\prime }$ is not BLDCM and there is no integrated encoder; this will also affect ${③}^{\prime }$, the choice of controller. For example, if voltage and current sensors are not integrated within the controller, then ⑨ and ⑩ are necessary or should be replaced with equivalent components.

*(2) Fault seed setup.*

**Figure 8:**
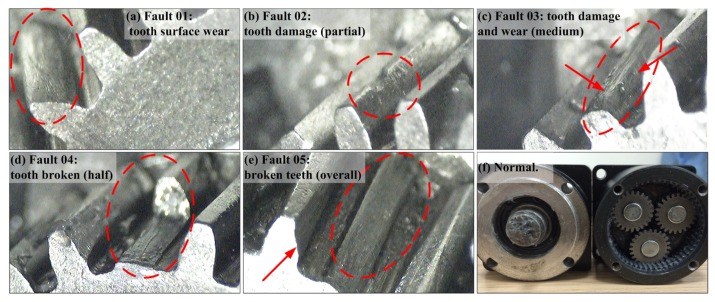
Faults of the planet reducer (zoom ×50).

Five types of faults were defined from mild to severe, as shown in Figure 8. Each type of gear fault was created by artificial damaging. Table 1 gives the test conditions and pattern labels. Both the healthy gearbox and the gearboxes with seeded faults were tested at seven different input shaft speeds. In addition, a 50-h rack operation was employed to simulate the compounding of multiple problems in the actual environment. For example, Figure 8c displays a combination of tooth damage and tooth surface wear. The adjacent teeth of the problem tooth marked in Figure 8e also have a certain degree of wear: originally, the pressure of the tooth sharing was borne by its proximity.

*(2) DAQ system:*

The sampling period of the first part was chosen at 20 Hz, as recommended for the motor controller shown in Figure 7a. In the second part, the speed and vibration frequency of each gearbox gear can be calculated [8]:

(14) $$f\left(s\right)=\omega_{1}\times s_{1}+\omega_{2}\times s_{2}+\cdots +\omega_{n}\times s_{n}$$

Figure 8f shows a specific PGB with a standstill ring gear that was used in this paper. The sun gear $z_{1}$ and the three planet gears $z_{2}$ had 15 teeth, and the ring gear $z_{3}$ had 45 teeth. For this type of PGB, the number of teeth was linear to the radius of each gear pitch circle. This fact indicates that the gear ratio was also related to the angular velocity ω of the gears. The gear ratio can be defined as:

(15) $$R=\omega_{1}/\omega_{A}=1+z_{3}/z_{1}$$

where $\omega_{i}$ is *R* the angular velocity of the *i*th gear component; $z_{i}$ is the number of teeth on the *i*th gear component; and the gear component index subscripts 1, 2, 3, and *A* correspond to sun gear, planet gear, ring gear, and arm (i.e., planet carrier), respectively. The planet carrier rotation speed (i.e., output shaft speed) in terms of frequency could be obtained as:

(16) $$f_{a}=f_{1}/R$$

where $f_{i}$ is the rotation speed in frequency at the *i*th gear component. In addition, a meshing characteristic frequency of PGB can be obtained as:

(17) $$f_{12}=f_{23}=f_{1}z_{1}z_{3}/\left(z_{1}+z_{3}\right)=f_{1}z_{3}/R$$

where $f_{ij}$ is the relative rotation speed in frequency between the *i*th and *j*th gear components.

The most common three failure modes of a PGB are the sun gear fault, planet gear fault, and ring gear fault. Their corresponding fault frequencies are represented as follows:

(18) $$\left\{\begin{array}{c} f_{f,1}=s\left(f_{1}-f_{a}\right)=f_{1}z_{3}s/\left(z_{1}+z_{3}\right)\\ f_{f,2}=2\left(f_{2}+f_{a}\right)=4f_{1}z_{1}z_{3}/\left(z_{3}^{2}-z_{1}^{2}\right)\\ f_{f,3}=sf_{a}=f_{1}z_{1}s/\left(z_{1}+z_{3}\right) \end{array}\right.$$

where $f_{f,i}$ represents the fault frequency at the *i*th gear component and *s* represents the number of planet gears in the gearbox. For more details, see [10]. Table 2 presents the structural information and characteristic frequencies of the PGB used in this paper.

We can determine the minimum effective sampling rate of the system through the above calculation. Data acquisition was performed in conjunction with the sensor shown in Figure 7. Table 3 displays the DAQ parameter setting. The DAQ system consisted of two parts: (1) BLDC integrated control system with a low sample rate and accuracy, integrating current, voltage, and rotary encoders. The basic control cycle is also a sampling rate of 20 Hz, although the maximum sampling period is only 1 kHz. The other part is (2) an NI DAQ board with a maximum analog input sampling rate of 1.25 MHz that integrates current and voltage sensors, two IEPE accelerometers, a torque sensor, and an encoder. Both parts were accessed via the MATLAB Data Acquisition Toolbox in order to obtain a uniform measurement time stamp. The BLDC and NI DAQ used the CAN (COM-CAN) and USB interface, respectively.

At each loading condition, 42 sample sets (42 × 9 sensors = 378 items) were taken. In addition, the system was preheated for one hour of continuous operation at 16 ${}^{\circ }$C in order to obtain accurate data.

### 5.2. Data Collection and Segment

*(1) Acquisition of raw datasets:*

Data acquisition was by DAQ as described in Section 5.1. Here, the main vibration sensor was taken as an example to describe the data and the subsequent dataset.

**Figure 9:**
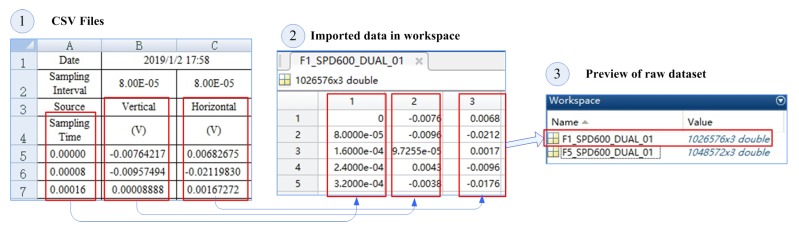
Acquisition of raw datasets.

① The raw data format was an Excel${}^{®}$-compatible Comma-Separated Values (CSV) file, as shown in Figure 9. It contained two parts: data description and data. It can be seen that the absolute sampling time of the sample was 1 February 2019, 17:58; and the sampling interval of both sensors was 8.00 $\times \;10^{-5}$ s, converted to frequency as 12.5 kHz. The data section was divided into three columns: sampling time relative to absolute time; Sensor 1 (vertically mounted); and Sensor 2 (horizontally mounted).

② Import raw data into processing tools such as MATLAB${}^{®}$. Two data files “F1_SPD600_DUAL_01” and “F5_SPD600_DUAL_01” were imported as a case study. Its naming convention was “[Fault type]-[input speed]-[sensor channel (single/dual channel)]-[sample number]”. That is, the sample failure types we imported were F1 and F5, respectively, and the speed was 600RPM, using vertical and horizontal dual-channel sensors.

③ We can preview the data to see their basic properties after import. As you can see, the imported data types were “double”, and the dataset dimensions were 1,026,576 × 3 and 1,048,572 × 3. Although the sampling time of these two sets of samples was about 100s, the amount of data was very large. We needed to align and split and align because the count of data rows 1,026,576 and 1,048,572 was different.

*(2) Data segmentation process:*

**Figure 10:**
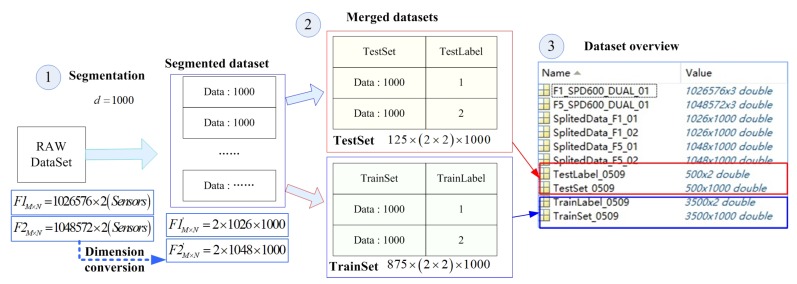
Data segement.

In order to support common deep learning methods, the original data needed to be segmented, merged, and tagged to form a training set and a test set. This process is shown in Figure 10.

① It is unlikely that the above raw dataset will be sent directly to the training model, even if we know the type of failure the sample belongs to; it is too large and does not conform to the basic idea of TSA. It is challenging to have a single run cycle for each sample segment during data segmentation. We do not know or it is difficult for ordinary users to know the start time and period of the operation. We only need to know that each fragment contains fault features, which is the benefit of the deep learning method. The size of the data split is usually $2^{n}\times 2^{n}$. Too small and too large a size will increase the training overhead. Additionally, $2^{4}\times 2^{4}=1024$ is a typical size. We defined the split size as 1000 to simplify the split process. For example, the dataset formed by the original dataset “F1_SPD600_DUAL_01” was divided into $SegmentedSet_{\left[rows,cols\right]}=2\left(\mathrm{sensors}\right)\times 1026\times 1000$ and 576 data that could not be divisible were discarded. ‘F5_SPD600_DUAL_01‘” was divided into $SegmentedSet_{\left[rows,cols\right]}=2\left(\mathrm{sensors}\right)\times 1048\times 1000$.

② The multiple divided dataset can be obtained to perform similar processing on all samples. These datasets need to be merged and tagged before they are sent to model training and testing. The split ratio used in this paper was 87.5%:12.5%. Thus, we obtained a test set size of 875×(2(faulttype)×2(sensors))×1000 and a training set of 125×(2(faulttype)×2(sensors))×1000. We set a fault label for each line of samples such that “TrainLabel” was 3500×1000 and “TestLabel” was 500×1000.

③ Through the processing of the above steps, we had a complete dataset that could be used directly for $test_{1}∼test_{4}$ testing, including the training set, training set label, test set, and test set label.

*(3) Data demonstration:*

**Figure 11:**
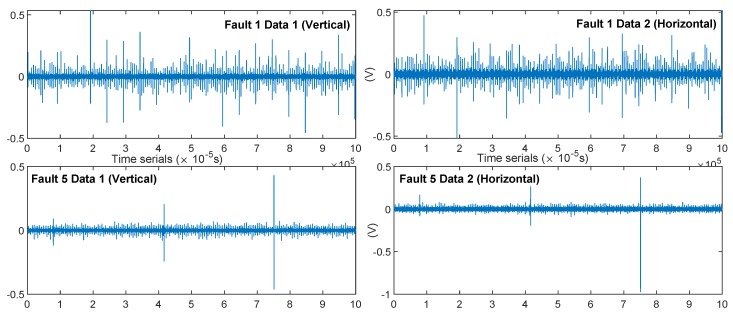
Comparison of the original waveform.

The time series comparison of the two samples “F1_SPD600_DUAL_01” and “F5_SPD600_DUAL_01” is shown in Figure 11. The waveform of the raw data provided the basic features: ① $F_{1}$ was not “clear” and contained more interference and signal components than $F_{5}$; ② both sensors in $F_{5}$ showed three high-intensity, periodic shock signals. It was difficult for us to find such periodic features in the sequence of F1, making it possible to distinguish between the two fault features, because the $F_{1}$ and $F_{5}$ fault seeds were significantly different.

**Figure 12:**
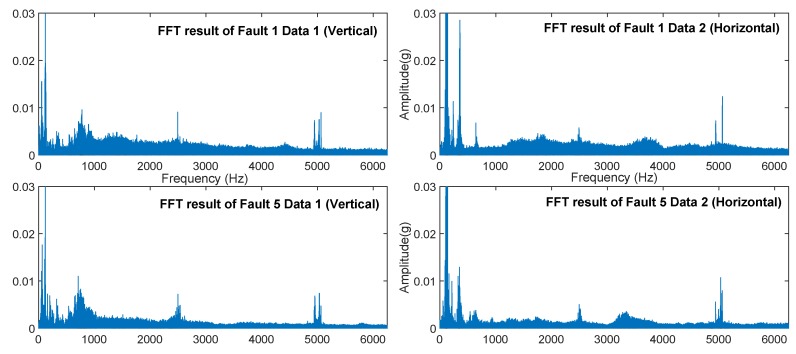
Comparison of the FFT waveform.

Converting the signal into a spectrum through FFT allowed us to understand the characteristics of the signal from another angle as shown in Figure 12 (the peak of the spectrum was suppressed to 0.03(g) in order to facilitate the observation of the details). We can observe that the primary frequencies had similar distribution characteristics because they had the same input speed and fault position in planetary gears. This is the raw signal shown in Figure 11 that cannot be provided directly. $F_{1}$ had higher energy (also including noise level) compared to $F_{5}$.

**Figure 13:**
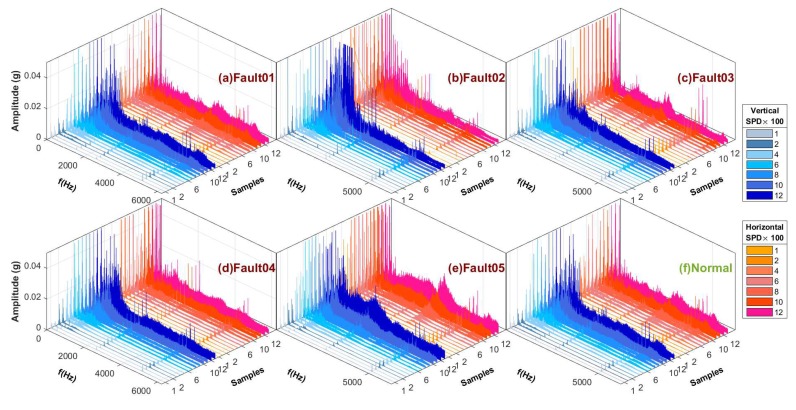
Spectrum comparison of six data sources.

We performed FFT conversion and visualization on the vibration signals of all six fault types and eight speed conditions based on the same processing method. Figure 13 displays the six types of sample spectra after FFT transformation that were acquired by the acceleration sensor (the peak of the spectrum was suppressed to 0.05(g) in order to facilitate the observation of details).

### 5.3. Test Group Setup

The data from each sensor were input as a feature parameter ($F_{1}$–$F_{9}$, as shown in Table 3) into the HDBN model based on the analysis and assumptions in Section 4.2. All or a part of the features were selected to perform the test, grouping them into data union and feature fusion. The grouping situation is shown in Table 4:

## 6. Results and Analysis

### 6.1. Results Analysis and Discussion of Different Test Groups

**Figure 14:**
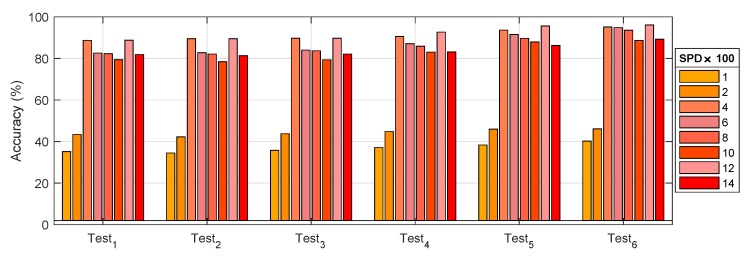
Recognition accuracy comparison of different test groups.

**Figure 15:**
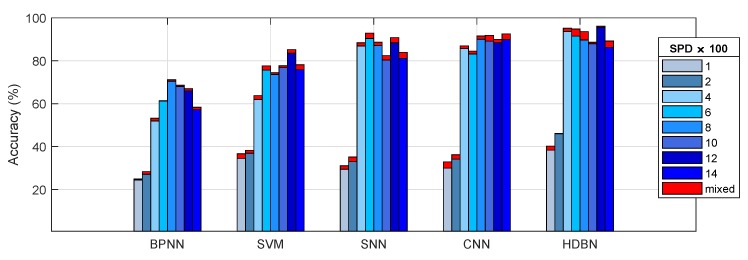
Recognition accuracy comparison of different models.

The recognition accuracy comparison of different test groups is shown in Figure 14. The test based on the grouping conditions shown in Table 4 was based on HDBN. The test results are shown in Table 5 and Figure 14. It can be seen that:

(1) The $test_{3}$ accuracy was 73.51% in the three types of fusion tests, that is the overall effect of the ⊎ method was the highest.
(2) $test_{4}$ and $test_{6}$ worked best in conditions that could be merged. By comparison, the weight of the vibration data from $F_{4}$ and $F_{5}$ contributed more than 80%. Other data, such as current and voltage, accounted for less than 3%. In addition, an average increase of 2.04% occurred based on $F_{4}^{\prime }$ and $F_{5}^{\prime }$ conversion from $\mathrm{FFT}(F_{4})$ and $\mathrm{FFT}(F_{5})$. It is worth noting that mixed precision training contributed an average of 1.88%. Hybrid data fusion and mixed precision training could effectively improve the accuracy of the model without relying on new data.
(3) Figure 15 displays the results of the group test. It can be observed that $Test_{6}$ had the highest accuracy. The accuracy of the difference between $test_{1}$ and $test_{2}$ was reduced, although the difference between $test_{1}$ and $test_{2}$ was small. The reason was that the accuracy of the model depended more on the number of samples if the total number of samples was not increased, although from a different fusion perspective.

### 6.2. Results Analysis and Discussion of Different Diagnostic Models

Based on the grouping test, the ⊎ method was selected as the benchmark data fusion method. In addition, a set of experiments was implemented that compared some state-of-the-art methods in the field of fault diagnosis by using the same sample set. The average classification accuracies of all the diagnostic models are listed in Table 6. The diagnostic performance of the model based on HDBN with deep feature learning was superior relative to the diagnostic model based on the shallow feature learning architecture, such as the SVM. However, the diagnostic performance of a model based on HDBN with multiple hidden layers was still unsatisfactory, especially at low-speed operating conditions. In addition, CNN achieved better accuracy in some tests such as the SPD1000 and SPD14000 conditions.

### 6.3. Results Analysis and Discussion of Fault Prediction

**Figure 16:**
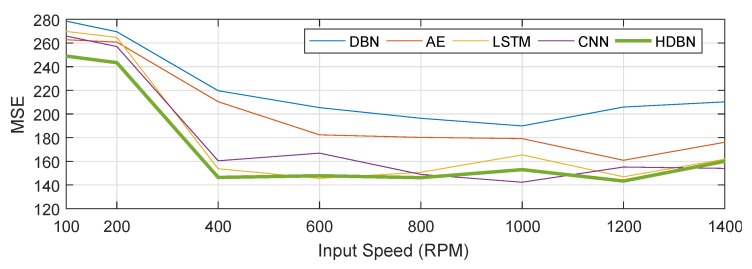
MSE comparison of fault prediction.

The fault prediction accuracy of various methods was compared based on MSE as shown in Table 7. Here, we selected $F_{5}$ fault seed, based on the test group $test_{6}$ and seven input speed conditions. Figure 16 displays the results of failure prediction.

After the basic comparison test was completed, we established a hybrid precision training test for each model. As shown in Figure 15, the mixed-precision training could improve the accuracy of the model to a certain extent, with an average increase of 1∼3%. By comparison, we can conclude the following:

(1) The neural networks with multiple hidden layers can preferably learn representative features from input data. By directly using the RMB algorithm to train multiple hidden layers, HDBN can easily fall into local optima, so that the performance is unstable. This shortcoming occurs because the initial weights and the deviation occurring in the process of error back propagation will affect the stability of neural networks.
(2) Compared with standard neural networks with multiple hidden layers, deep learning consists of two procedures: unsupervised pre-training and supervised fine-tuning. Deep learning can effectively solve the problem of local optima by using unsupervised pre-training layer-by-layer to find the optimal initial weights before fine-tuning these weights.
(3) The diagnostic model based on HDBN can automatically and adaptively learn deep features and the complex nonlinear relevance between the input data of the model and fault patterns. The performance of the model is less dependent on prior knowledge and diagnostic experience.
(4) In the MSE error analysis of fault prediction, HDBN still achieved good performance. However, it should be noted that the error of the above method was large under low speed conditions. The waveform characteristics of the system at higher speeds in the verification system environment were more significant, and the (noise) energy level was also lower, as can be seen from Figure 11 and Figure 12. The effect of noise at low speeds was significant.

## 7. Conclusions

In this work, we proposed to exploit data sources and the implications of their integration to better extract depth features when building diagnostic models. First, a deep fault feature learning method was established based on DBN, and the hybrid data fusion method was used to establish the HDBN diagnostic model. Second, three data fusion methods were established according to the type and physical meaning of the signal instead of the “data union or data mix” used in the existing research. Experiments showed that different fusion methods were related to the actual characteristics of the data, and the appropriate fusion method and dataset composition method could improve the learning accuracy. Third, mixed-precision training was used as a special data fusion method to further improve the performance of the model. Finally, the experimental results confirmed that the deep learning and ⊎ data fusion of HDBM effectively improved the performance of intelligent fault diagnosis and outperform the other diagnostic models. In the future, the author will further study the problem of fault diagnosis in complex environments, including optimized diagnostic methods under multi-load and multi-drive input crosstalk conditions.

## Figures and Tables

**Table 1:** Pattern labels and test conditions of the gear fault.

Pattern Label	Gearbox Condition	Input Speed (RPM)	Load
1	Tooth surface wear	100, 200, 400, 600, 800, 1200, 1400	Null
2	Tooth damage (partial)		Null
3	Tooth damage (medium)		Null
4	Tooth broken (half)		Null
5	Broken teeth (overall)		Null
6	Normal		Null

**Table 2:** Characteristic frequencies of the PGB at varied input shaft speeds (Hz).

Input Shaft Speed (RPM)	Output Shaft Speed ($f_{a}$)	Meshing Frequency ($f_{12}=f_{23}$)	Sun Gear Fault Frequency ($f_{f,1}$)	Planet Gear Fault Frequency ($f_{f,2}$)	Ring Gear Fault Frequency ($f_{f,3}$)
100	0.42	18.75	3.75	2.5	1.25
200	0.83	37.50	7.50	5.0	2.50
400	1.67	75.00	15.00	10.0	5.00
600	2.50	112.50	22.50	15.0	7.50
800	3.33	150.50	30.00	20.0	10.00
1000	4.17	187.50	37.50	30.0	12.50
1200	5.00	225.00	45.00	35.0	15.00
1400	5.83	262.50	52.50	35.0	17.50

**Table 3:** Sensor parameter setting.

Part	Sensor	Feature	Values	Weight	Rate
(2)	Current	$F_{1}$	50 mA	$w_{1}$	20 Hz
(2)	Voltage	$F_{2}$	100 mV	$\omega_{2}$	
(2)	Encoder	$F_{3}$	1000PPR	$\omega_{2}$	
(6)	$IEPE_{V}$	$F_{4}$	102 mV/g	$w_{4}$	12.5 kHz
(6)	$IEPE_{H}$	$F_{5}$	98 mV/g	$\omega_{4}$	
(7)	Torque sensor	$F_{6}$	0.01 N·m	$\omega_{5}$	
(8)	Encoder	$F_{7}$	1024 PPR	$\omega_{6}$	
(9)	Current	$F_{8}$	50 mV/A	$\omega_{7}$	
(9)	Voltage	$F_{9}$	98.7 mV/V	$\omega_{8}$	
(10)	A/D converter		16 bit	$\omega_{9}$	

**Table 4:** Test grouping settings.

Test Group	Grouping Method	Test Group	Grouping Method
$Test_{1}$	$F_{4}\cup F_{5}$	$Test_{4}$	$\left(F_{4}\cup F_{5}\right)⊎\left(F_{4}⊎F_{5}\right)⊎\left(F_{4}^{\prime }\cup F_{5}^{\prime }\right)$
$Test_{2}$	$F_{4}⊕F_{5}$	$Test_{5}$	$Test_{4}\cup F_{1}\cdots \cup F_{3}\cup F_{6}\cdots \cup F_{9}$
$Test_{3}$	$F_{4}⊎F_{5}$	$Test_{6}$	$Test_{5}\cup \mathrm{Mixed}\_\mathrm{precision}\left(F_{4},F_{5}\right)$

**Table 5:** Group test results using HDBN.

Speed (RPM)	${\mathrm{Test}}_{1}$ (%)	${\mathrm{Test}}_{2}$ (%)	${\mathrm{Test}}_{3}$ (%)	${\mathrm{Test}}_{4}$ (%)	${\mathrm{Test}}_{5}$ (%)	${\mathrm{Test}}_{6}$ (%)
100	35.14	34.51	35.75	37.13	38.34	**40.21**
200	43.38	42.23	43.75	44.85	45.95	**46.09**
400	88.73	89.47	89.76	90.56	93.64	**95.16**
600	82.56	82.77	84.01	87.12	91.52	**94.83**
800	82.29	82.12	83.65	85.91	89.67	**93.61**
1000	79.42	78.46	79.34	83.08	87.93	**88.67**
1200	88.78	89.47	89.73	92.67	95.59	**96.13**
1400	81.87	81.32	82.11	83.10	86.25	**89.22**
average	72.77	72.54	73.51	75.55	78.61	**80.49**

**Table 6:** Comparison of different models under $test_{6}$.

Speed (RPM)	SVM (%)	BPNN (%)	DBN (%)	CNN (%)	HDBN (%)
100	24.89	36.67	31.09	32.86	**40.21**
200	28.34	38.21	35.14	36.22	**46.09**
400	53.18	63.77	88.38	86.96	**95.16**
600	61.34	77.62	92.91	84.50	**94.83**
800	71.14	74.46	88.62	91.58	**93.61**
1000	68.58	77.66	82.41	**91.87**	88.67
1200	66.96	85.19	90.81	89.99	**96.13**
1400	58.36	78.15	83.92	**92.53**	89.22
average	54.1	66.47	74.16	75.81	**80.46**

**Table 7:** MSE comparison results of the main methods in failure prediction.

Method	100	200	400	600	800	1000	1200	1400
DBN	278.49	269.52	219.69	205.39	196.38	189.93	205.88	210.27
AE	262.76	260.77	210.38	182.38	180.24	179.2	160.89	176.13
LSTM	269.79	264.73	153.73	**145.62**	150.75	165.43	146.94	161.81
CNN	265.87	257.02	160.47	166.91	148.97	**142.34**	155.2	**154.06**
**HDBN**	**248.96**	**243.38**	**146.42**	147.82	**146.2**	152.98	**143.35**	160.03

## Abbreviations

The following abbreviations are used in this manuscript:

A/D	Analog-to-Digital Converter
AC	Alternating Current Power
AE	Autoencoder
AM-FM	Amplitude Modulation and Frequency Modulation
ANFIS	Adaptive Neuro-Fuzzy Inference System
BLDCM	Brushless Direct Current Motor
CF	Condition Factor
CIs	Condition Indicators
CM	Confusion Matrix
CNNs	Convolutional Neural Networks
COM-CAN	Communication Interface-Controller Area Network Converter
CSV	Comma-Separated Values
DAE	Denoising Auto-Encoder
DAQ	Data Acquisition
DBMs	Deep Boltzmann Machines
DBN	Deep Belief Network
DC	Direct Current Power
DL	Deep Learning
DNN	Deep Neural Network
ELMD	Ensemble Local Mean Decomposition
EO	Energy Operator
FFT	Fast Fourier Transform
FT	Fourier Transform
GKPCA	Greedy Kernel Principal Component Analysis
HDBN	Hybrid Deep Belief Network
IEPE	Integrated Electronics Piezoelectric
k-CDM	K-Step Contrastive Divergence Method
KT	Kurtosis
LSTM	Long Short-Term Memory
MAE	Mean Absolute Error
MHMS	Machine Health Monitoring Systems
NN	Neural Network
P2P	Peak-to-Peak
PCA	Principal Component Analysis
PGB	Planetary Gearboxes
PKFA	Probabilistic Kernel Factor Analysis
RMBs	Restricted Boltzmann Machines
RMS	Root Mean Square
RNNs	Recurrent Neural Networks
SAE	Stacked Auto-Encoder
SAE-DBN	Sparse Autoencoder-Deep Belief Network
SAEs	Sparse Auto-Encoders
SCADA	Supervisory Control and Data
SD	Standard Deviation
SDAs	Stacked Denoising Automatic encoders
SK	Skewness
TSA	Time Synchronous Averaged
WPE	Wavelet Packet Energy
